# Inhibition of Autophagy Facilitates XY03-EA-Mediated Neuroprotection against the Cerebral Ischemia/Reperfusion Injury in Rats

**DOI:** 10.1155/2022/7013299

**Published:** 2022-03-30

**Authors:** Wenwen Cui, Yuanyuan Hao, Mingye Wang, Qiuyan Zhang, Junmei Wang, Gang Wei, Yunlong Hou

**Affiliations:** ^1^College of Integrated Chinese and Western Medicine, Hebei University of Chinese Medicine, Shijiazhuang 050035, China; ^2^New Drug Evaluation Center, Shijiazhuang Yiling Pharmaceutical Co., Ltd, Shijiazhuang 050035, China; ^3^National Key Laboratory of Collateral Disease Research and Innovative Chinese Medicine, Shijiazhuang 050035, China; ^4^Shijiazhuang Compound Traditional Chinese Medicine Technology Innovation Center, Shijiazhuang 050035, China

## Abstract

**Objective:**

L-3-n-Butylphthalide (NBP) is used to treat moderate and severe acute ischemia stroke. A previous screening study indicates that XY03-EA, a novel derivative of NBP, is more potent than NBP in the oxyradical scavenging capacity. In this study, *in vivo* and *in vitro* ischemia/reperfusion (I/R) models were used to test whether the XY03-EA offered therapeutic benefits in the ischemic stroke and explore the underlying mechanism of action.

**Methods:**

For this purpose, behavioral scores, cerebral infarct volume, cerebral blood flow, oxidative stress levels, inflammatory factor expression, energy metabolism levels, and autophagy activation were estimated in the rat middle cerebral artery occlusion and reperfusion (MCAO/R) model. The nonhuman primate MCAO/R model was conducted to validate the therapeutic effect of XY03-EA applied for 3 weeks. The neurological deficit score (NDS) progression rate and the infarct volume were continuously recorded on days 3, 7, 14, and 21. The PC-12 cell OGD/R model was used to assess the cell survival rate, reactive oxygen species (ROS) levels, the expression of autophagy execution molecules, and the activation of autophagy-related signaling pathways.

**Results:**

XY03-EA decreased the cerebral injuries and NDS by increasing cerebral blood flow, improving brain energy metabolism, accelerating ROS clearance, suppressing inflammatory responses, and inhibiting autophagy in the MCAO/R model rats. In the nonhuman primate MCAO/R model, the treatment of XY03-EA for 3 weeks could significantly inhibit the NDS progression rate and indicate a positive trend to reduce the infarct volume in a dose-dependent way. Mechanistically, XY03-EA inhibited ROS-dependent autophagy activation and thereby protected the PC-12 cells from the autophagic cell death induced by OGD/R.

**Conclusions:**

In this study, we found that XY03-EA alleviated the cerebral I/R injuries in rats and nonhuman primates. Our results demonstrated that XY03-EA exerted neuroprotective effects against the ROS-mediated autophagic neurocyte death and had great potential for the treatment of ischemic stroke.

## 1. Introduction

Although the morbidity, mortality, and recurrence rates of stroke have decreased over the past few decades, stroke is still the second leading cause of death and contributor to disability globally [1]. It kills roughly 5.5 million people and affects the health of about 13.7 million people annually, causing huge economic losses worldwide [2]. Ischemic stroke caused by thrombotic plaques accounts for approximately 87% of strokes, causing brain cell death and severe tissue damage [3, 4]. The key factors leading to cell death include oxidative stress, inflammation, energy depletion, homeostasis loss, acidosis, intracellular calcium level increase, excitatory toxicity, free radical-mediated toxicity, cytokine-mediated cytotoxicity, and blood-brain barrier damage [5–7]. At present, stroke treatment mainly focuses on restoring cerebral blood flow by intravenous thrombolysis and rapid reperfusion of thrombectomy [8, 9]. However, reperfusion therapies must be administered within a narrow time window, thereby drastically limiting the eligible patients. There is a continuing need for the development of novel drugs tailored to target the pathogenetic mechanisms underlying ischemic injuries.

Despite blood flow recovery in the cerebral ischemic region, further tissue and microcirculation damages still occur, which is called ischemia/reperfusion (I/R) injury [10]. Reactive oxygen species (ROS) are an important participant in the I/R injury [11]. When the yield of ROS caused by I/R injury exceeds the scavenging activity, a large number of cells will die, leading to excessive tissue damage [12]. Numerous studies have shown that ROS can activate autophagy through a variety of different mechanisms [11]. Autophagy has been widely accepted to play an important role in maintaining intracellular homeostasis by the removal of damaged organelles and unfolding proteins [13]. However, it remains uncertain whether autophagy plays a protective role in ischemic stroke. Generally, moderate autophagy can control the intracellular ROS level to contribute to maintaining intracellular ROS homeostasis, thereby protecting the cerebral tissue and cell against I/R injury [14]. In contrast, excess autophagy occurs, possibly activated by accumulating ROS levels in I/R injury, which may lead to neuronal cell death and aggravate tissue damage [15]. Thus, the role of autophagy in ischemic stroke is still controversial and may exert both protective and/or detrimental effects as a “double-edged sword.” Notably, recent studies reveal that compound K [16], epicatechin gallate [17], and N-acetyl-L-cysteine [18] inhibit autophagy-induced cell death by scavenging ROS. Here, we proposed that scavenging ROS reduced the autophagic cell death by inhibiting excess autophagy in cerebral I/R injury.

L-3-n-Butylphthalide (NBP), also known as levo-apigenin extracted from celery seed, has been used in the treatment of mild to moderate acute ischemic stroke by improving blood circulation and protecting nerve tissue [19–21]. Studies on the MOA have shown that NBP attenuates nerve cell injury via blockage of intracellular ROS production and downregulation of oxidative stress levels [22, 23]. Meanwhile, NBP has also been shown to protect against brain injury by modulating the level of intracellular autophagy [24, 25]. Further studies are needed to shed more light on the role of ROS scavengers in the treatment of ischemic stroke via autophagy regulation. Here, we demonstrated a novel compound, XY03-EA, derived from the NBP structure, which had been proved to exert a better scavenging capacity on ROS (Supplementary data 1). Our previous data verified that XY03-EA exerted better effects on improving SOD activity and GSH content and reducing the content of MDA in the brain tissue of I/R injured.

**Figure 1:**
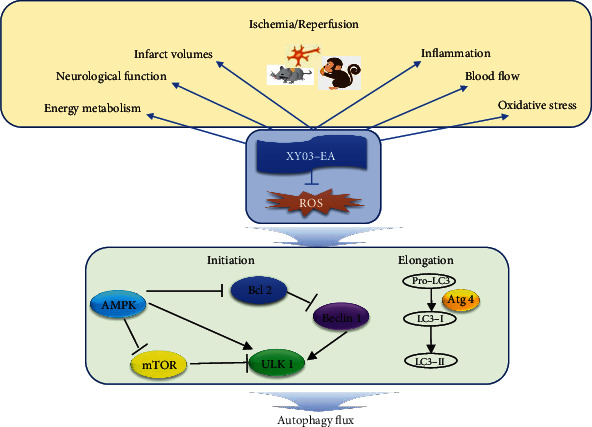
Summary graphic illustration: XY03-EA protects the brain tissue and cell vitality from I/R injury in neural cells, rodents, and nonhuman primates. The autophagic cell death caused by excessive ROS is inhibited by XY03-EA by scavenging ROS. ROS: reactive oxygen species; AMPK: adenosine 5′-monophosphate-activated protein kinase; Bcl-2: B-cell lymphoma-2; mTOR: mammalian target of rapamycin; ULK1: UNC-51-like kinase 1; LC3: microtubule-associated protein-1 light chain 3; Atg: autophagy-related proteins.

In this study, as shown in Figure 1, we comprehensively evaluated the pharmacological effect of XY03-EA in both rodent and primate animal models of I/R. To gain mechanistic insight on the effect of XY03-EA in the protection of neurons, we demonstrated that XY03-EA inhibited autophagy to scavenge the excessive ROS caused by I/R injury. These results provided a new option for the clinical treatment of ischemic stroke.

## 2. Materials and Methods

### 2.1. Compounds

**Figure 2:**
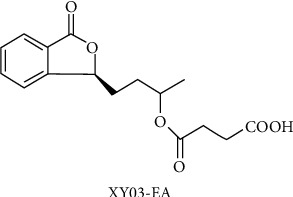
Chemical structure of XY03-EA

The compounds used are NBP (Lot: 118161109, purchased by Shijiazhuang Pharmaceutical Co. Ltd, Shijiazhuang, Hebei Province, China) and XY03-EA (Figure 2) (molecular weight is 306.11, Lot: 170320, provided by Shijiazhuang Yiling Pharmaceutical Co. Ltd, Shijiazhuang, Hebei Province, China).

### 2.2. Animals

Adult male Sprague-Dawley rats weighing 180–200 g were provided from the Vital River Laboratory Animal Technology Co. Ltd (Certificate No. SYXK (Jing) 2016-0006, Beijing, China). The rats were maintained under standard laboratory conditions that contained a 12 h-12 h natural dark-light cycle and a temperature-controlled environment (20–26°C) in cages with 5 rats per cage, and the standard maintenance diet (Jiangsu Synergy Pharmaceutical Bioengineering Co. Ltd) for rats and pure water were available ad libitum. After 3-7 days of adaptive feeding, the rats were randomly divided into 7 groups when their body weight reached 220-240 g. The experimental procedures and animal welfare were in accordance with the Ethics Review Committee for Animal Experimentation of New Drug Evaluation Center of Hebei Yiling Medical Research Institute.

Adult male rhesus monkeys aged 3-6 years old were provided from the Sichuan Greenhouse Biotechnology Co. Ltd (Certificate No. SCXK (Chuan) 2014-013, Chengdu, China). The monkeys were maintained in the general monkey areas that contained a 12h-12h natural dark-light cycle and a temperature-controlled environment (17.93–24.70°C) in double stainless steel monkey cages with one monkey per cage, and the standard maintenance diet (Beijing Keaoxieli Feed Co. Ltd) for monkeys, some fruits, and reverse osmosis water were available ad libitum. The experimental procedures and animal welfare were in accordance with the Ethics Review Committee for Animal Experimentation of Chengdu Huaxihaiqi Medical Technology Co. Ltd.

### 2.3. Experimental Groups and Drug Treatment

Rats were divided into the following seven groups: control group (control), Sham operation group (Sham), MCAO/R model group (MCAO/R), MCAO/R+NBP 62 mg/kg group (NBP), MCAO/R+XY03-EA 25 mg/kg group (XY03-EA 25 mg/kg), MCAO/R+XY03-EA 50 mg/kg group (XY03-EA 50 mg/kg), and MCAO/R+XY03-EA 100 mg/kg group (XY03-EA 100 mg/kg). The dose of the NBP group was the isomolar dose of the XY03-EA100 mg/kg group. The administration volume was 5 mL/kg by oral gavage. The concentration of XY03-EA was 20 mg/mL, 10 mg/mL, and 5 mg/mL, and the concentration of NBP was 12.4 mg/mL with 0.5% CMC-Na, and rats of the control group, Sham group, and MCAO/R group were given 0.5% CMC-Na. Rats of all groups were given a single intragastric administration 10 minutes after ischemia.

Monkeys were divided into the following four groups: MCAO/R model group (MCAO/R), MCAO/R+XY03-EA 25 mg/kg group (XY03-EA 25 mg/kg), and MCAO/R+XY03-EA 50 mg/kg group (XY03-EA 50 mg/kg). There were 6 monkeys per group in the MCAO/R group and the XY03-EA 50 mg/kg group and 4 monkeys per group in XY03-EA 25 mg/kg group. Oral gavage administration was given for the first time about 2 h after the completion of surgical modeling (when the animals were fully awake) and then once a day for 20 consecutive days. The administration volume was 5 mL/kg. The drug preparation method was the same as the rat study.

Rat experiments were carried out three times. The first rat experiment was used to detect the neurological deficits and cerebral infarct volume of rats. There were 12 rats per group in the control group and the Sham group and 20 rats per group in the treatment groups (MCAO/R group, NBP group, XY03-EA 25 mg/kg group, XY03-EA 50 mg/kg group, and XY03-EA 100 mg/kg group). The second rat experiment was used to detect the brain energy metabolism, antioxidant capacity, inflammation, and cerebral blood flow. There were 10 rats per group in the control group and Sham group and 14 rats per group in the treatment groups. The third rat experiment was used to detect the mitochondrial membrane potential. There were 8 rats per group in the control group and Sham group and 12 rats per group in the treatment groups.

### 2.4. Middle Cerebral Artery Occlusion and Reperfusion (MCAO/R) Model

**Figure 3:**
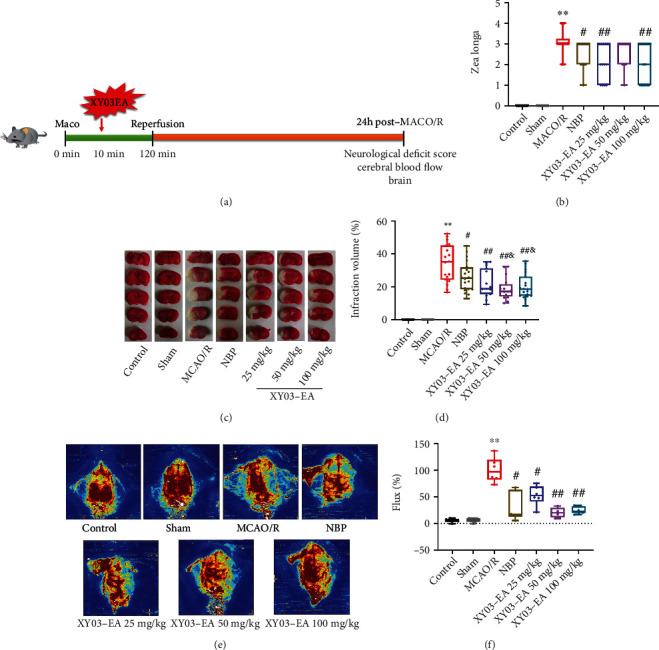
Effect of XY03-EA on NDS, infarct volumes, and blood flow in rats after MCAO/R. (a) Timeline illustration of rat experiments. (b) Neurological deficit evaluation was conducted after MCAO/R (first experiment), the higher score represents a more serious deficit (*n*: control and Sham groups = 12, other groups = 20). (c) Representative TTC staining images of the brain slices after MCAO/R. White area: infarct area; red area: normal area. (d) Infarction range was quantified by TTC staining started from the frontal cortex to the occipital cortex (*n*: control and Sham groups = 12, other groups = 20). (e) Representative blood flow images of the brain in rats after MCAO/R. (f) Flux (%) was quantified by blood flow measured with a laser Doppler flowmetry probe (*n* = 6). ^∗∗^*p* < 0.01 vs. the Sham group; ^#^*p* < 0.05 and ^##^*p* < 0.01 vs. the MCAO/R group; ^&^*p* < 0.05 vs. the NBP group.

Based on the classic rat MCAO cerebral ischemia model as previously described [26, 27], we made a slight modification to establish the rat MCAO/R model to induce ischemic brain injury. The MCAO silica gel bolted threads (L3600 or L3800, Guangzhou Jialing Biotechnology Co. Ltd) were irradiated with UV for 30min for use. In brief, rats were anesthetized with isoflurane (4% induction and 2% maintenance), fixed in supine position, and disinfected with iodophor. The incision was 4-5 cm long along the midline of the neck. The right common carotid artery (CCA), external carotid artery (ECA), internal carotid artery (ICA), the branch of the superior thyroid artery (ST) issued by ECA, and the occipital artery (OA) from ICA bifurcation were separated. And ST and OA were cut off by an electrocoagulation pen to prevent bleeding. After temporarily clamping the CCA and ICA with the microvascular clips, the distal end of ECA was ligated and a small incision was cut 4 mm away from the bifurcation of CCA. The prepared thread was pushed from the external carotid artery to the internal carotid artery. The threads were pushed gently with the ophthalmic forceps and fixed the bolt position when there was slight resistance, and the insertion depth was about 20 mm. After 2 h of ischemia, reperfusion was achieved by slowly withdrawing out the threads. In the rats of the Sham group, the right common carotid artery area with the same method was exposed without other treatments. The control group did not receive any surgical intervention. After 2 h ischemia and 24 h reperfusion, the rats were euthanized and brain tissues were collected (Figure 3(a)).

**Figure 4:**
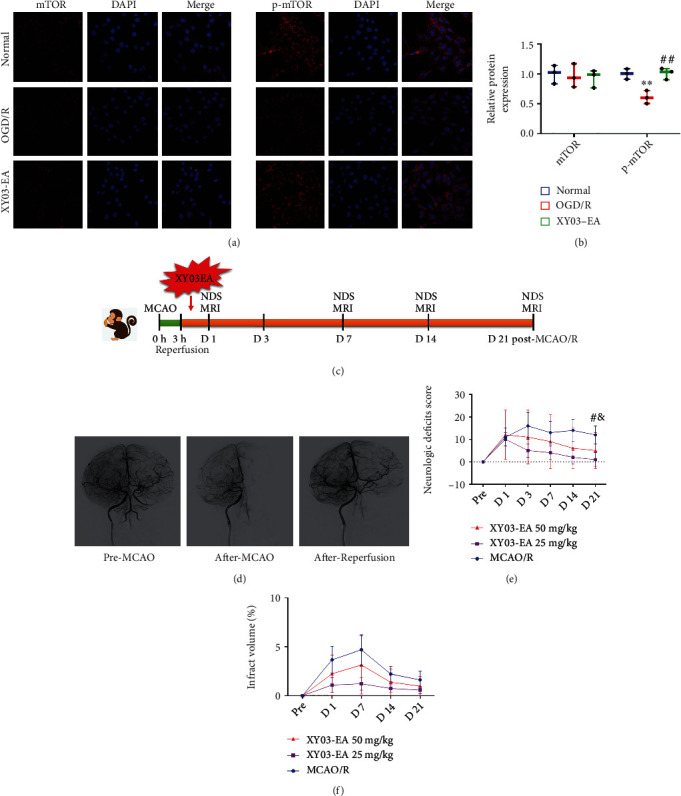
Effect of XY03-EA on NDS and infarct volumes in monkeys after MCAO. (a) mTOR and p-mTOR proteins in PC-12 cells were detected by immunofluorescence. Scale bar: 20 *μ*m, ×400. (b) Fluorescence intensity of mTOR and p-mTOR protein was analyzed by ImageJ (*n* = 3). ^∗∗^*p* < 0.01 vs. the normal group; ^#^*p* < 0.05 vs. the OGD/R group. (c) Timeline graph of the rhesus monkey experiments. (d) DSA images of the brain were obtained before and after the MCAO model and reperfusion in rhesus monkeys. (e) Neurological deficit evaluation was conducted at 1 day, 3 days, 7 days, 14 days, and 21 days after MCAO/R in monkeys. ^#^*p* < 0.05, the XY04-EA 25 mg/kg group vs. the MCAO/R group; ^&^*p* < 0.05, the NBP group vs. the MCAO/R group. (f) Infarction range was quantified by MRI at 1 day, 7 days, 14 days, and 21 days after MCAO/R in monkeys.

In this study, a middle cerebral artery occlusion/reperfusion (MCAO/R) model was established by minimally invasive intervention in rhesus monkeys [28]. In brief, rhesus monkeys were fasted for 12 hours before anesthesia. After 0.1 mL/kg intramuscular injection of Shutai 50 (6F13/67TE, Vic Zoletil, France) anesthesia, the monkeys were fixed in the supine position on the operating table. The right groin was prepared routinely. The strongest point of the right femoral artery pulsation was taken as the puncture point, and the 5 F artery sheath (RS∗A50K10SQ, Terumo, Japan) was inserted. The vascular sheath was placed in the right internal carotid artery, and a 5 F angiography catheter (Envoy, Johnson, USA) was inserted under the guidance of the guide wire to conduct the digital subtraction angiography (DSA). Under the guidance of DSA, the microcatheter (105-5091-150, EV3, USA) was directed to the middle and distal right middle cerebral artery (RMCA) and then, the detachable spring coil (NC-1.5-2-HELIX, EV3, USA) was released, which completely blocked the blood. Observe the development of RMCA. The contrast agent in the microcatheter with a pressure syringe was injected to perform superselective angiography (Allura Xper FD20, Philips, Netherlands). After confirming the RMCA block, the spring ring was placed there, and the blood flow was blocked for 3 hours. Then, the spring coil was withdrawn, and the superselective angiography was performed again to confirm the blood recovery of RMCA. The first dose was given about 2 hours after the completion of surgical modeling (after the animal was fully awake) and then once a day for 20 consecutive days (a total of 21 days). The infarct volume of the brain was measured by the MRI at D1, D3, D7, D14, and D21. The respiratory rate, blood oxygen, and heart rate of animals were monitored by the monitor (BeneView T8, Mindray, China) in real time during operation (Figure 4(c)).

### 2.5. Assessment of Neurological Deficit Score (NDS)

After cerebral infarction for 24 h, the behavior of experimental rats was evaluated by the Zea Longa neurological function scoring method [29]. The criteria were used as follows: 0 = no neurological deficits, 1 = failure to extend the right forepaw fully, 2 = circling to the right, 3 = paresis to the left, 4 = no spontaneous walking, and 5 = death or loss of consciousness.

Before the MCAO/R modeling and after the MCAO/R modeling for D1, D3, D7, D14, and D21, the behavior of experimental monkeys was evaluated by the following method (Table 1): the NDS was scored out of 100 on a scale of 0 to 28 for state of consciousness, 0 to 32 for motor system, 0 to 18 for skeletal muscle coordination, and 0 to 22 for sensory system.

(1) $$\begin{array}{c} \text{The rate of NDS progression }\left(\%\right)=\frac{\left(\text{score}-{\text{score}}_{\text{D}1}\right)}{{\text{score}}_{\text{D}1}}*100\%. \end{array}$$

### 2.6. 2, 3, 5-Triphenyltetrazolium Chloride (TTC) Staining and Infarct Volume Measurement of the Rats

After evaluating the neurological deficit in the first experiment, the rats were killed under deep anesthesia and the brain tissues were removed. After removing the olfactory bulb, cerebellum, and lower brainstem, the brain tissues were frozen in the refrigerator for 15 minutes and divided into 5 slices by 4-knife coronary artery resection. Brain slices were stained with 1.2% 2,3,5-triphenyltetrazolium chloride solution (TTC, Sigma-Aldrich, USA) in a dark constant temperature water bath at 37°C about 20 minutes.

TTC staining showed that the normal brain tissues were rose red and the infarcted brain tissues were white. Brain slices were placed on the filter paper in order and taken pictures. And then, the infarcted brain tissues were carefully dug out and weighed. The infarct volume (%) was calculated as the percentage of infarct tissue weight in the operation side. The inhibition rate (%) of each treatment group was calculated according to the infarction area of the operated side. The calculation formula is as follows:

(2) $$\begin{array}{c} \text{The inhibition rate }\left(\%\right)=\frac{\left({\text{weight}}_{\text{MCAO}/\text{R}}-{\text{weight}}_{\text{treatment}}\right)}{{\text{weight}}_{\text{MCAO}/\text{R}}}*100\%. \end{array}$$

### 2.7. Infarct Volume of the Monkey Measured by Magnetic Resonance Imaging (MRI)

Before the MCAO/R modeling and after the MCAO/R modeling for D1, D3, D7, D14, and D21, the infarct volume of the brain was measured by the MRI (MAGNETOM Skyra 3T, Siemens, Germany). Monkeys were general anesthetized by intravenous injection of 2.5% sodium pentobarbital and fixed on the scanning bed. The scanning sequence of MRI consisted of T1WI (T1-weighted imaging), T2WI (T2-weighted imaging), T2-FLAIR (liquid attenuation inversion recovery), and DTI (diffusion tensor imaging): T1WI: repeat time (TR) = 1500 ms, echo time (TE) = 10 ms, scan layer thickness = 2 mm, scan spacing = 0.4 mm, scan layer number = 24 layers, flip angle = 150°, matrix 256 × 216, field of vision (FOV) 130 mm × 109.7 mm; T2WI: TR = 5500 ms, TE = 108 ms, scan layer thickness = 2 mm, scan spacing = 0.4 mm, scan layer number = 24 layers, flip angle = 150°, matrix 320 × 270, FOV = 130 mm × 109.7 mm; T2-FLAIR: TR = 6000 ms, TE = 79 ms, scan layer thickness = 2 mm, scan spacing = 0.4 mm, scan layer number = 24 layers, flip angle = 150°, matrix 192 × 168, FOV = 130 mm × 113.8 mm; DTI: TR = 4400 ms, TE = 85 ms, scanning layer thickness = 2 mm, scanning spacing = 0.4 mm, scanning layer number = 24 layers, matrix 116 × 116, FOV = 158 mm × 158 mm.

The presence of infarct lesions was confirmed based on T1WI, T2WI, T2-FLAIR, DTI, and MRA scan results, and the infarct volume was calculated based on T2WI images. The infarct volume was calculated using ITK-SNA.P software and the area superposition method. In brief, the infarct area of 24 cross-sections was added up and multiplied by the layer thickness of 2 mm to obtain the infarct volume and the whole brain volume, respectively. The ratio of the infarct volume and the whole brain volume was the percentage of the infarct volume in the whole brain volume.

### 2.8. Measurement of Regional Cerebral Blood Flow (CBF) of the Rats

After evaluating the neurological deficit in the second rat experiment, CBF during cerebral ischemia was measured with a laser Doppler flowmetry probe (Moor Instruments Ltd). In brief, under anesthesia, a small incision was made in the center of the skull. Remove the underlying fascia and press it until there was no bleeding point on the skull. A small area of the skull was exposed to allow placement of the laser-Doppler probe.

The calculation formula is as follows: flux (%) = flux_ischemic_/flux_contralateral_∗100%.

### 2.9. Measurement of Mitochondrial Membrane Potential of the Rats

Mitochondrial membrane potential in rat brain tissues was qualitatively assessed by JC-1 Assay Kit (C2006, Beyotime, China). Single cell suspension of the brain tissue was prepared by trypsin digestion method: ischemic brain tissue was taken and placed in 0.125% trypsin digestion. Fetal bovine serum was added to stop digestion, and the supernatant was centrifuged to obtain single brain cell suspension. The procedure was performed in accordance with the manufacturer' protocol. Mitochondrial membrane potential was measured by fluorescence method using a microtiter plate reader (Tecan Infinite M200 Pro, Switzerland). When detecting JC-1 monomer, the excitation and emission light were 490 nm and 530 nm respectively. When detecting JC-1 polymer, the excitation and emission light were 525 nm and 590 nm respectively.

### 2.10. Measurement of Oxidative Stress, Inflammation, and Energy Metabolism of the Rats

Superoxide dismutase (SOD), malondialdehyde (MDA), and glutathione (GSH) are markers of oxidative stress; interleukin- (IL-) 1*β*, IL-6, and tumor necrosis factor- (TNF-) *α* are markers of inflammation; and ATP and lactic acid (LD) are markers of energy metabolism. The ischemic tissue samples were prepared into tissue homogenate in sodium phosphate buffer (PBS) containing proteinase inhibitor phenylmethylsulfonyl fluoride (PMSF). The supernatant was collected after 3000-4000 × g centrifugation for 15 min at 4°C, and the total protein content was detected by BCA kit (Beyotime, Shanghai, China). The levels of SOD, MDA, GSH, ATP, and LD in the brain tissue were measured according to the Nanjing Jiancheng Bioengineering manufacturer's instructions (SOD: A001-3, MDA: A003-1, GSH: A006-2, and ATP: A016-2, LD: A019-2). 2-(4-Iodophenyl)-3-(4-nitrophenyl)-5-(2,4-disulfophenyl)-2H-tetrazolium, monosodium salt (WST-1) method is used to detect the activity of total SOD. MDA is detected by thiobarbituric acid (TBA) method. GSH can react with dithiodinitrobenzoic acid (DTNB) to produce a yellow compound, and the absorbance value is measured at 405 nm to calculate the content of GSH. Lactate dehydrogenase (LDH) colorimetry is used to detect the content of LD. ATPase can decompose ATP to produce ADP and inorganic phosphorus. The activity of ATPase can be judged by measuring the amount of inorganic phosphorus. The levels of IL-1*β*, IL-6, and TNF-*α* in the brain tissue were measured according to the instructions of Abcam ELISA kit (IL-1*β*: ab100768, IL-6: ab119548, and TNF-*α*: ab100785). Absorbance values were determined by measuring luminescent signal the by using a microtiter plate reader (Tecan Infinite M200 Pro, Switzerland).

### 2.11. Pharmacokinetic Study of XY03-EA on MCAO/R Rats

After MCAO modeling and administration (as described above), the whole blood of rats in XY03-EA administration groups (25, 50, and 100 mg/kg) was collected at different time points (before administration, 10 min, 1 h, 2 h, and 4 h after administration). Eight male SD rats were selected from each group. The whole blood was collected from 0.15 mL to 1.5 mL EP tubes. Immediately, 50 *μ*L of the whole blood samples was collected into 1.5 mL EP tubes containing 400 *μ*L acetonitrile solution (including 80 nM senkyunolide H) with pipetting device, then mixed with vortex for 5 s, and stored at -70°C. The brain tissue samples of rats were the XY03-EA administration group (100 mg/kg, 2 h after administration), and the brain tissue samples of 8 male SD rats were divided into the ischemic area and contralateral side. The weighed rat brain tissue was thawed at 5°C, cut into small pieces, homogenized with a 19-fold 50% CH3CN solution, and stored at -70°C.

Liquid chromatography-mass spectrometry combined analysis system (LC/MS/MS) was composed of a German Agilent 1290 Infinity II series liquid chromatography analyzer and API4000 QTrap mass spectrometry detector. The operating software of the system was 1290 II (HPLC) and Analyst (mass spectrometry). A validated LC/MS/MS method was applied to determine the concentration of XY03-EA in biological samples. Pharmacokinetic parameters of XY03-EA including area under concentration–time curve (AUC), mean residence time (MRT), *C*_max_, *T*_1/2_, and *T*_max_ were calculated using non-av models using InnaPhase Kinetica 2000™ software (USA).

### 2.12. Cell Culture

PC-12 cells were obtained from the Shanghai Cell Bank of Chinese Academy of Sciences (Shanghai, China) and were cultured in Dulbecco's Modified Eagle's Medium (DMEM, Gibco, Invitrogen, USA) supplemented with heat-inactivated 10% (*v*/*v*) FBS (Gibco, Invitrogen, USA), and 1% (*v*/*v*) penicillin and streptomycin (ThermoFisher Scientific, USA). Cells were maintained in a fully humidified, 5% CO_2_ incubator (Thermo 3111, USA) at 37°C. When cells were approximately 80% confluent, the cells were digested with 0.25% trypsinase (Gibco, Invitrogen, USA) and subcultivated at a ratio of 1 : 2-1 : 4 every 2 to 3 days.

### 2.13. Oxygen-Glucose Deprivation and Reoxygenation (OGD/R) Model Construction

To establish the OGD/R injury model, the PC-12 cells were seeded in 96-well plates or six-well plates at a certain concentration. When the density of cells reached 40-50%, they were rinsed three times with PBS and the medium and conditions were changed. Firstly, cells of the treatment groups were incubated in a three-gas hypoxic incubator (Nuaire, NU-5741E, USA) in serum- and glucose-free DMEM at 37°C for 16 h in a humidified atmosphere of 1% O_2_, 5% CO_2_, and 94% N_2_. Then, cells were restored to normal culture condition in the normoxic incubator (5% CO_2_ and 95% air) for 8 h to establish reoxygenation.

### 2.14. Cell Viability Assays

In order to evaluate the effect of XY03-EA under normal culture conditions and its protective potential against OGD/R model conditions on PC-12 cells, the cell viability was evaluated by using the CellTiter-Glo® Luminescent Cell Viability Assay (G7572, Promega, USA) according to the manufacturer's protocol. In brief, PC-12 cells were seeded at a density of 10^4^ cells per well in 96-well white plates and cultured to adhere for 24 h. After the treatment of different concentrations of XY03-EA or culture conditions, the cells were added the CellTiter-Glo® reagent that has been reconstituted in advance for 2 minutes on an orbital shaker to induce cell lysis and incubated at room temperature for 10 minutes to stabilize luminescent signal. Cell viability was determined by measuring luminescent signal the by using a microtiter plate reader (Tecan Infinite M200 Pro, Switzerland).

### 2.15. Western Blot

Total proteins from brain tissues and PC-12 cells were extracted by RIPA lysis buffer with 0.1% PMSF (P0013C, Beyotime, China). BCA protein assay kit (P0012, Beyotime, China) detected total protein concentration. The homogenized protein samples were fractionated by 4-20% precast gel (M42015C/M42010C GenScript, China) at 120 V for 1.5 h and transferred onto nitrocellulose filter (NC) membranes (Bio-Rad Laboratories, Hercules, USA) at 110–120 V for 1 h. The membranes were blocked in blocking buffer (Beyotime, Shanghai, China) for 15-30 min at room temperature, followed by an overnight incubation at 4°C with primary antibodies (Anti-LC3B, ab192890, 1 : 1000; Anti-SQSTM1/P62, #5114, 1 : 1000; Anti-Atg4B, ab154843, 1 : 1000; Anti-Bcl-2, ab59348, 1 : 1000; Anti-Beclin 1, ab207612, 1 : 1000; Anti-AMPK*α*(D5A2), #5831, 1 : 1000; Anti-p-AMPK*α*(Thr172)(40H9), #2535, 1 : 1000; Anti-ZO-1, ab96587, 1 : 500; Anti-*β*-actin, #3700, 1 : 1000) and respective fluorescent secondary antibodies (Goat anti-Mouse IgG H&L IRDye® 680RD, ab216776, 1 : 5000; Goat anti-Rabbit IgG H&L IRDye® 800CW, ab216773, 1 : 5000) at 37°C for 1-2 h. Finally, the immunoreactive bands were visualized using Odyssey CLx fluorescence scanning system (LI-COR Biosciences, USA). Equal protein loading was normalized with Anti-*β*-actin antibody.

### 2.16. Immunofluorescent Staining

For *in vivo* studies, after MCAO/R for 24 h, rats were deeply anesthetized and the brains were removed after cardiac perfusion with ice-cold 0.9% sodium chloride injection. Then, the brains were rapidly embedded in Sakura Tissue-Tek OCT Compound (4583, Sakura, USA) and sectioned into slices of 10 *μ*m thickness. For *in vitro* studies, the cells were cultured in clear glass plates and fixed with 4% formaldehyde for 20 minutes after ODG/R and treatment. Brain tissue sections and fixed PC-12 cells were permeabilized with 0.5% Triton X-100 for 10 minutes and blocked for 60 minutes; the primary antibody (Anti-LC3B, ab192890, 1 *μ*g/mL; Anti-SQSTM1/P62, ab109012, 1 *μ*g/mL; Anti-mTOR, #2983, 1:200; Anti-p-mTOR, #5536, 1 : 50) was incubated overnight at 4°C, and the fluorescent secondary antibody (P0179 and P0176, Beyotime, China) was incubated for 60 minutes in the dark. After being washed three times, add mounting solution with DAPI. PC-12 cells were transfected with Premo™ autophagy tandem sensor RFP-GFP-LC3B kit (Cat. P36239, ThermoFisher Scientific, USA) according to the manufacturer's protocol. Cells were counterstained with Hoechst solution and fixed with 4% formaldehyde. The images were collected using a confocal microscope (LSM710, Zeiss, Germany) and analyzed with ImageJ.

### 2.17. Total Reactive Oxygen Species (ROS) and Superoxide Assessments

ROS and superoxide levels in the live cells were measured using a commercial kit (ab139476, Abcam, USA). The procedure was performed in accordance with the manufacturer' protocol. Upon staining, the fluorescent products generated by the two dyes can be visualized using a wide-field confocal microscope (LSM710, Zeiss, Germany) equipped with standard green (Ex/Em = 490/525nm) and orange (Ex/Em = 550/620nm) filter set and by cytometry using any a flow cytometer (FACSAria III, BD, USA) equipped with a blue laser (488 nm filter).

### 2.18. Statistical Analysis

All of the data are presented as mean ± SD. All analyses were performed using SPSS 19.0 statistical software. The statistics and data evaluation were subjected to statistical analysis using Levene homogeneity of variance test first. When variances were homogeneous (*p* > 0.05), the results of one-way analysis of variance (ANOVA) were used to determine whether the overall difference was statistically significant. When variances were not homogeneous (*p* ≤ 0.05), Kruskal-Wallis *H* test was used to show whether the overall difference was statistically significant. ^#^*p* < 0.05, ^##^*p* < 0.01, ^∗^*p* < 0.05, and ^∗∗^*p* < 0.01 were considered significant.

## 3. Results

### 3.1. Effect of XY03-EA on NDS in Rats after MCAO/R

The rats in the control group and the Sham group had normal behavior and activity with a score of 0. Compared with the Sham group, the neurological function of the MCAO/R group was significantly damaged, and the Zea Longa scores significantly increased (*p* < 0.01). Compared with the MCAO/R group, the Zea Longa scores of the treatment groups significantly decreased (*P* < 0.05, *P* < 0.01), except that the XY03-EA 50 mg/kg group showed a decreasing trend (Figure 3(b)).

### 3.2. Effect of XY03-EA on Infarct Volumes in Rats after MCAO/R

There was no infarction in the brain tissue of the control group and the Sham group. Compared with the Sham group, the infarct volumes of the operation side of the MCAO/R group significantly increased (*p* < 0.01). Compared with the MCAO/R group, the infarct volumes of all treatment groups significantly reduced (*p* < 0.05, *p* < 0.01). Compared with the NBP group, the infarct volumes of the XY03-EA 50 mg/kg and 100 mg/kg groups were significantly decreased (*p* < 0.05) (Figures 3(c) and 3(d)).

### 3.3. Effect of XY03-EA on the Blood Flow in Rats after MCAO/R

The blood flow of the rats in the control group and the Sham group was almost identical on the ischemic and contralateral sides. Compared with the Sham group, the cerebral blood flow of rats in the MCAO/R group significantly decreased (*p* < 0.01). Compared with the MCAO/R group, the cerebral blood flow of rats in all treatment groups significantly improved (*p* < 0.05, *p* < 0.01) (Figures 3(e) and 3(f)).

### 3.4. Effect of XY03-EA on Mitochondrial Membrane Potential and Energy Metabolism in Rats after MCAO/R

**Figure 5:**
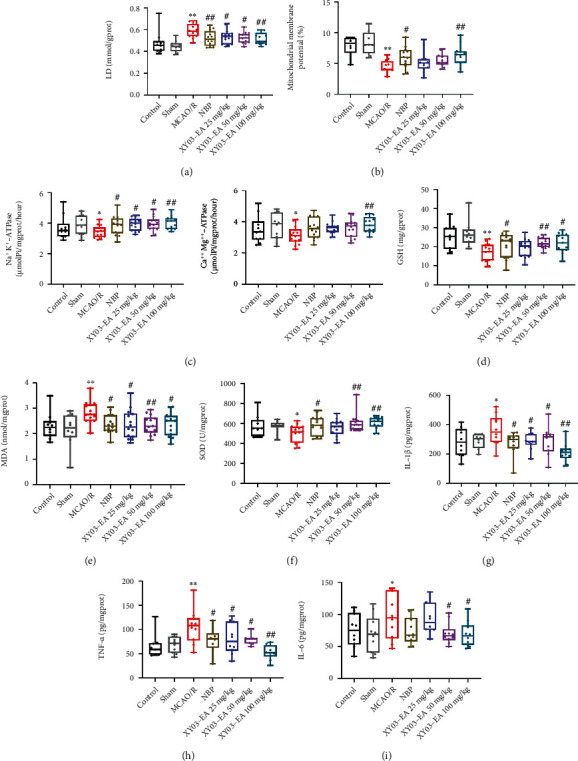
Effect of XY03-EA on oxidative stress, inflammation, and energy metabolism in rats after MCAO/R. (a, c) Energy metabolism (LD, Na^+^K^+^-ATPase and Ca^2+^Mg^2+^-ATPase) in rats after MCAO/R (*n*: control and Sham groups = 10, other groups = 14). (b) Mitochondrial membrane potential in rats after MCAO/R (*n*: control and Sham groups = 8, other groups = 12). (d–f) Oxidative stress (GSH, MDA, and SOD) in rats after MCAO/R (*n*: control and Sham groups = 10, other groups = 14). (g–i) Inflammatory factors (TNF-*α*, IL-1*β*, and IL-6) in rats after MCAO/R (*n* = 10). ^∗^*p* < 0.05 and ^∗∗^*p* < 0.01 vs. the Sham group; ^#^*p* < 0.05 and ^##^*p* < 0.01 vs. the MCAO/R group.

Compared with the Sham group, the energy metabolism level in brain tissue of the MCAO/R group decreased. It showed that the activity of Na^+^K^+^-ATPase and Ca^2+^Mg^2+^-ATPase decreased significantly (*p* < 0.05), while the content of LD was increased significantly (*p* < 0.01), and the mitochondrial membrane potential of the brain tissue was significantly decreased (*p* < 0.01). Compared with the MCAO/R group, the activity of Na^+^K^+^-ATPase significantly increased and LD content significantly decreased in all treatment groups (*p* < 0.05 or *p* < 0.01), and the mitochondrial membrane potential was significantly increased in the XY03-EA 100 mg/kg and NBP groups (*p* < 0.05, *p* < 0.01) (Figures 5(a)–5(c)).

### 3.5. Effect of XY03-EA on Oxidative Stress and Inflammation in Rats after MCAO/R

Compared with the Sham group, the oxidative stress and inflammatory response increased. It showed that there was a strong trend for SOD activity to decrease (*p* = 0.054) and the content of GSH decreased significantly (*p* < 0.01). And the content of MDA, TNF-*α*, IL-1*β*, and IL-6 were significantly increased (*p* < 0.05 or *p* < 0.01). Compared with the MCAO/R group, XY03-EA reduced oxidative stress and inflammation in the brain tissues of rats after MCAO/R. It showed that the SOD activity and GSH content in the NBP, XY03-EA50 mg/kg and 100 mg/kg groups significantly increased, and the content of MDA, TNF-*α*, and IL-1*β* in all treatment groups significantly decreased (*p* < 0.05 or *p* < 0.01), and IL-6 content of XY03-EA 50 mg/kg and 100 mg/kg group significantly reduced (*p* < 0.05) (Figures 5(d)–5(i)).

### 3.6. Pharmacokinetic Characteristics of XY03-EA on MCAO/R Rats

After MCAO/R modeling and XY03-EA was taken orally lavage at the dosages of 25, 50, and 100 mg/kg, it was rapidly absorbed with a peak plasma concentration time of 0.4-0.8 hours and quickly eliminated (half-life 2-5 hours). The *C*_max_ of XY03-EA (25, 50, and 100 mg/kg) in the whole blood were 2710 ± 4569, 2647 ± 2924, and 14174 ± 16787 nM, and AUC_0-4h_ were 1672 ± 1633, 2022 ± 1467, and 6960 ± 7170 nM·h, respectively. The exposure level of XY03-EA in rats (AUC_0-4h_ and *C*_max_) was positively correlated with dosage. After MCAO/R modeling and oral administration of XY03-EA (100 mg/kg) for 2 hours, the exposure level of XY03-EA was 3740 ± 1224 ng/g in the ischemic area brain tissue and 2940 ± 1030 ng/g in the contralateral brain tissue (Table 2).

### 3.7. Effect of XY03-EA on Expression of Autophagy-Related Proteins in the Brain Tissue after MCAO/R

**Figure 6:**
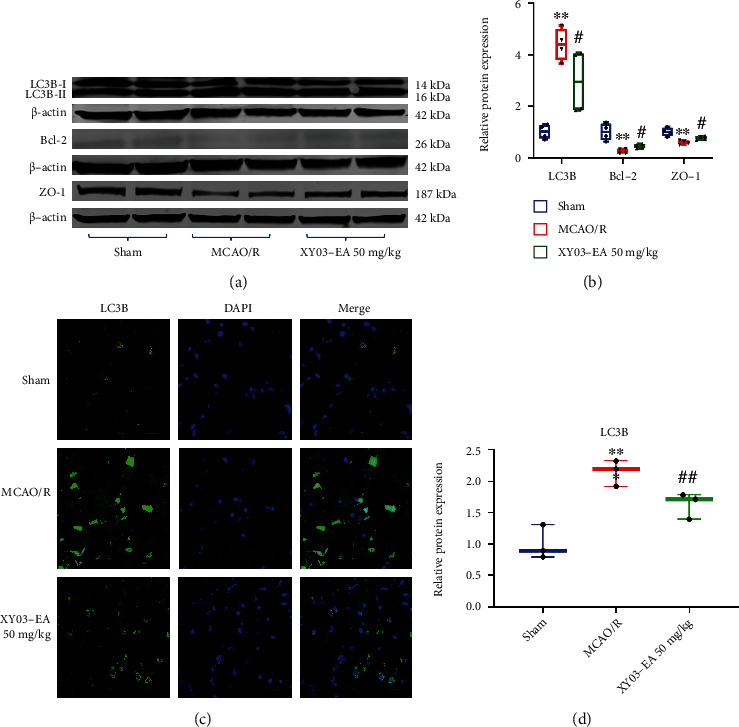
Effect of XY03-EA on expression of proteins in the brain tissue after MCAO/R. (a) Proteins of Bcl-2, LC3B, ZO-1, and *β*-actin in the brain tissue after MCAO/R were analyzed by Western blot. (b) Bands of interest were normalized against *β*-actin, and relative protein expression was provided by bands of Western blot (*n* = 4). (c) LC3B protein in the brain tissue after MCAO/R was detected by immunofluorescence. Scale bar: 20*μ*m, ×400. (d) Fluorescence intensity of LC3B protein was analyzed by ImageJ (*n* = 3). ^∗^*p* < 0.05 and ^∗∗^*p* < 0.01 vs. the Sham group; ^#^*p* < 0.05 vs. the MCAO/R group.

Light chain 3 (LC3) is currently recognized as a marker for autophagy. In both results of Western blot and immunofluorescence, compared with the Sham group, LC3B expression in the MCAO/R groups significantly increased (*p* < 0.01). Compared with the MCAO/R group, LC3B expression in the XY03-EA group significantly decreased (*p* < 0.05, *p* < 0.01). Results of Western blot showed that the expression of Bcl-2 and ZO-1 in the MCAO/R group significantly decreased compared with that in the Sham group (*p* < 0.01), and compared with the MCAO/R group, the expression of ZO-1 and Bcl-2 in the XY03-EA group significantly increased (*p* < 0.05) (Figures 6(a)–6(d)).

### 3.8. Effect of XY03-EA on Survival Rate of PC-12 Cells under the Normal and OGD/R Conditions

**Figure 7:**
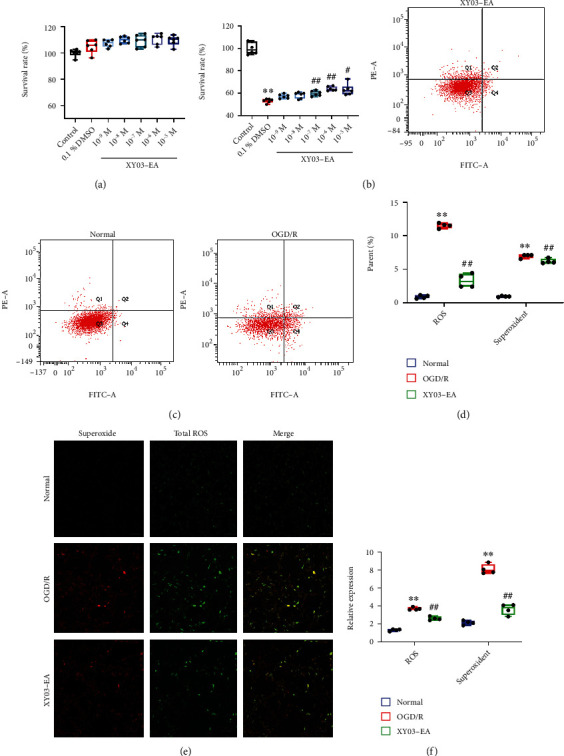
Effect of XY03-EA on survival rate and ROS expression of PC-12 cells after OGD/R. (a) Effect of XY03-EA on the survival rate of PC-12 cells under the normal conditions (*n* = 6). (b) Effect of XY03-EA on the survival rate of PC-12 cells under the OGD/R conditions (*n* = 6). (c–f) ROS expression of PC-12 cells was detected by a flow cytometer and confocal microscope (*n* = 4). Scale bar: 100 *μ*m, ×100. Oxidative stress detection reagent (green) for ROS detection and superoxide detection reagent (red). Signals produced by peroxides, peroxynitrite, and hydroxyl radicals were detected in the FL1 channel. Superoxide production was detected in the FL2 channel. ^∗∗^*p* < 0.01 vs. the control or normal group; ^#^*p* < 0.05 and ^##^*p* < 0.01 vs. the OGD/R group.

Under the normal culture condition, XY03-EA in the concentration range of 10^−5^-10^−9^ mol/L did not affect the survival rate of PC-12 cells. Under the OGD/R condition, XY03-EA increased the cell survival rate in the range of 10^−5^-10^−7^ mol/L in a concentration-dependent manner. This result suggested that XY03-EA can protect nerve cells from I/R injury. Therefore, 10^−6^ mol/L was selected for the follow-up *in vitro* study of XY03-EA (Figures 7(a) and 7(b)).

### 3.9. Effect of XY03-EA on ROS Expression of PC-12 Cells after OGD/R

Compared with the normal group, the expression of ROS and superoxide in the OGD/R group significantly increased (*p* < 0.01). Compared with the OGD/R group, the expression of ROS and superoxide significantly decreased in the XY03-EA group (*p* < 0.01, *p* < 0.05). The results suggested that XY03-EA can alleviate the oxidative stress injury of nerve cells induced by OGD/R (Figures 7(c)–7(f)).

### 3.10. Effects of XY03-EA on the Expression of Autophagy-Related Pathway Proteins in PC-12 Cells after ODG/R

**Figure 8:**
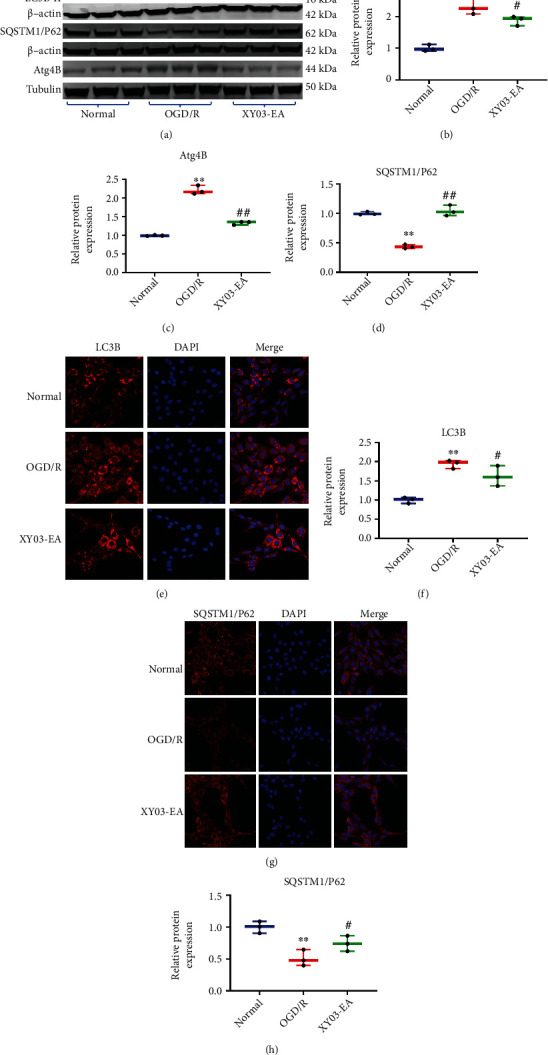
Effects of XY03-EA on the expression of autophagy-related proteins in PC-12 cells after ODG/R. (a) Proteins of LC3B, SQSTMI/P62, Atg5, Atg4B, and *β*-actin in PC-12 cells were analyzed by Western blot. (b–e) Bands of interest were normalized against *β*-actin, and relative protein expression was provided by bands of Western blot (*n* = 3). (f, h) LC3B and SQSTMI/P6 proteins in PC-12 cells were detected by immunofluorescence. Scale bar: 10 *μ*m (LC3B) and 20 *μ*m (SQSTMI/P62), ×400. (g, i) Fluorescence intensity of LC3B and SQSTMI/P6 protein was analyzed by ImageJ (*n* = 3). ^∗∗^*p* < 0.01 vs. the normal group; ^#^*p* < 0.05 and ^##^*p* < 0.01 vs. the OGD/R group.

Compared with the normal group, the expression of SQSTM1/P62 in the OGD/R group decreased, and the expression of LC3B-II/I and Atg4B in the OGD/R group increased (*p* < 0.01). Compared with the OGD/R group, the expression of SQSTM1/P62 significantly increased, and LC3B-II/I and Atg4 significantly decreased in the XY03-EA group (*p* < 0.01, *p* < 0.05) (Figures 8(a)–8(h)).

**Figure 9:**
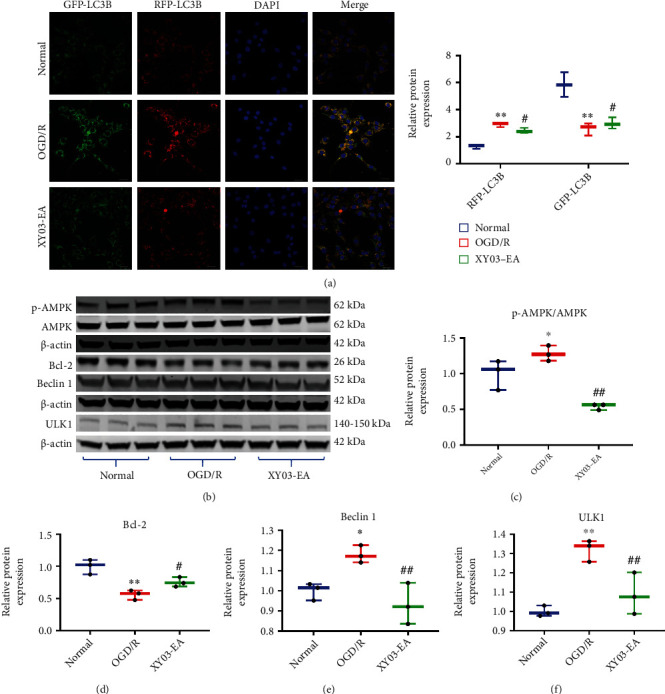
Effects of XY03-EA on the autophagosome maturation and expression of autophagy-related pathway proteins in PC-12 cells after ODG/R. (a) The appearance of red LC3B spots indicated that autophagosomes and lysosomes were successfully fused (*n* = 3). Scale bar: 20 *μ*m, ×400. The appearance of green LC3B spots indicated that the fusion of autophagosomes and lysosomes was affected. Compared with the control group, there were more red spots in the OGD/R group. Compared with the OGD/R group, there were less red spots in the XY03-EA group. ^∗∗^*p* < 0.01 vs. the normal group; ^#^*p* < 0.05 vs. the OGD/R group. (b) Proteins of p-AMPK, AMPK, Bcl-2, Beclin 1, ULK1, and *β*-actin in PC-12 cells were analyzed by Western blot. (c–f) Bands of interest were normalized against *β*-actin, and relative protein expression was provided by bands of Western blot (*n* = 3). ^∗^*p* < 0.05 and ^∗∗^*p* < 0.01 vs. the normal group; ^#^*p* < 0.05 and ^##^*p* < 0.01 vs. the OGD/R group.

Compared with the normal group, the expression of Beclin 1, ULK1, and p-AMPK/AMPK in the OGD/R group increased, and the expression of Bcl-2 and p-mTOR decreased (*p* < 0.01, *p* < 0.05). Compared with the OGD/R group, the expression of Bcl-2 and p-mTOR in the XY03-EA group significantly increased, while the expression of Beclin 1, ULK1, and p-AMPK/AMPK decreased (*p* < 0.01, *p* < 0.05) (Figures 9(b)–9(f), 4(a), and 4(b)).

### 3.11. Effects of XY03-EA on the Autophagosome Maturation in PC-12 Cells after ODG/R

The chimera of Premo™ Autophagy Tandem Sensor contained TagRFP and Emerald GFP proteins, complexed with LC3B. It can monitor autophagosome maturation, which is the process of LC3B from autophagy to autophagolysosome by tracing pH changes. The GFP-RFP sensor works on between acidic autolysosomes (PH = 4, RFP, red colour) and neutral autophagosomes (PH = 7, GFP, green colour). The appearance of red LC3B spots indicates that autophagosomes and lysosomes were successfully fused. The appearance of green LC3B spots indicates that autophagosomes and lysosomes cannot be fused. Compared with the normal group, there were more red spots in the OGD/R group. Compared with the OGD/R group, there were fewer red spots than in the XY03-EA group. It showed that XY03-EA inhibited the autophagosome and lysosome fusion of OGD/R PC-12 cells (Figure 8(a)).

### 3.12. Effect of XY03-EA on NDS and Infarct Volumes in Monkeys after MCAO/R

At 4 consecutive monitoring time points from 3 days to 21 days after surgery, NDS scores of monkeys in the XY03-EA 25 mg/kg group and XY03-EA 50 mg/kg group showed a significant downward trend, and the size of NDS was as follows:XY03 − EA 25 mg/kg group < XY03 − EA 50 mg/kg group < MCAO/R group. The NDS of the XY03-EA 25 mg/kg group was significantly lower than that of the MCAO/R group at 21 days after surgery (*p* = 0.05). At 21 days and 14 days after the operation, the NDS progression rate of the three treatment groups was significantly lower than that of the MCAO/R group (*p* < 0.05) (Figure 4(e), Table 3). XY03-EA improved the neurological function of nonhuman primate MCAO/R model animals.

The cerebral infarct volume was calculated by MRI scan. At each time point, the cerebral infarction volume of animals in the XY03-EA 25 mg/kg group was the lowest (0.57%-1.22%), followed by the XY03-EA 50 mg/kg group (0.98%-3.13%) and the MCAO/R group (1.62%-4.68%). There was no significant difference in cerebral infarct volume among the three drug treatment groups and the MCAO/R group (Figure 4(f)). But XY03-EA reduced the cerebral infarction area of nonhuman primate MCAO/R model animals, and it was dose-dependent.

## 4. Discussion

This study provided experimental evidence that the XY03-EA exerted neuroprotective effects on the rat MCAO/R model, which was also validated in the nonhuman primate MCAO/R models. Additionally, *in vitro* and *in vivo* studies implicated that the XY03-EA scavenged ROS to suppress oxidative stress injury. To gain mechanistic insight into the neuroprotective effects, XY03-EA was proved to inhibit the ROS-dependent activation of autophagy *in vivo* and *in vitro*.

To validate the neuroprotective effects of XY03-EA on I/R injury *in vivo*, the MCAO/R model in rats was established in this study. Compared with the rats in the MCAO/R model group, XY03-EA improved the NDS of MCAO/R injury by reducing the size of cerebral infarction, increasing the cerebral blood flow in the ischemic area, suppressing the oxidative stress injury and inflammatory response, improving the energy metabolism, and maintaining the integrity of the blood-brain barrier. To exclude the species differences in pharmacodynamics and more conform to the principles of clinical application, a nonhuman primate cerebral MCAO/R model was used to verify the efficacy of the use of XY03-EA for a long term in ischemic stroke. The results showed that XY03-EA could also reduce NDS score and promote neurological function recovery, confirming that XY03-EA had great potential as a candidate for further clinical studies in patients with ischemic strokes.

In ischemic stroke, disruption of blood supply to the brain tissue (ischemia), followed by reperfusion (reperfusion), causes ROS to “explode.” Oxidative stress is created by the excessive ROS that is not balanced by the endogenous enzymatic and nonenzymatic antioxidants, leading to widespread damages by oxidation of lipid acid, protein, and DNA, resulting in cell death [13, 14, 30]. The level of MDA, a product of lipid peroxidation, represents the level of oxidative stress in an organism. In contrast, the level of SOD and GSH indicates the body's ability to scavenge ROS and relieve oxidative stress [31, 32]. XY03-EA increased the content of GSH and the activity of SOD, inhibited the increase of MDA in the brain tissue of the ischemic region *in vivo* (Figures 5(d)–5(f)), increased the survival rate of PC-12 cells injured with OGD/R, and decreased the expression of intracellular ROS *in vitro* (Figures 7(a)–7(f)), which suggested that XY03-EA had a great potential in mitigating the oxidative stress injury by promoting the scavenging of ROS.

ROS, as a major source of damage in biological systems, irreversibly oxidizes DNA and cellular biomolecules. Besides, ROS regulates autophagy through multiple pathways; in turn, autophagy contributes to clearing the cells of all irreversibly oxidized biomolecules (proteins, DNA, and lipids) [13]. Thus, ROS cooperates with autophagy to maintain intracellular homeostasis [33]. Abundant evidence demonstrates that appropriate autophagy stimulates tissue repair by inhibiting oxidative stress and inflammation, while inappropriate or defective autophagy aggravates tissue damage during I/R injury [34]. When cerebral I/R occurs, an accumulation of ROS in the brain tissue activates autophagy, and autophagy acts as a buffer system to control intracellular ROS levels and relieve oxidative stress injury [35]. However, with the accumulation of ROS, defective or excessive autophagy might be induced, leading to autophagic cell death [36]. Due to the bidirectional actions of autophagy, the actual function of antioxidants on autophagy execution is still the subject of debate [13]. In this study, the results from the levels of MDA, SOD, and GSH in the rat brain tissue of the MCAO/R model indicated the increased level of oxidative stress. The expression of microtubule-associated protein-1 light chain 3 (LC3) B-II, a biomarker for the formation of autophagosome [37], was also significantly increased, indicating the activation of autophagy. We found that XY03-EA decreased the expression of LC3B-II/I in the rat brain tissues of the MCAO/R model (Figures 6(a)–6(d) and 5(d)–7(f)). These results indicated that XY03-EA *in vivo* scavenged oxygen free radicals and blocked the maturation of autophagosomes. In the OGD/R-induced I/R injury model of PC-12 cells *in vitro*, XY03-EA increased the survival rate of PC-12 cells and decreased the expression of intracellular ROS; in addition, XY03-EA significantly decreased the expression of LC3B-II/I and Atg4 and blocked the degradation of P62, one of the autophagy receptors/adaptors binding to LC3-II for the maturation of autophagosome (Figures 8(a)–8(h)). Using Premo™ autophagy tandem sensor RFP-GFP-LC3B transfected PC-12 cells, we found that XY03-EA inhibited the autophagosome and lysosome fusion (Figure 9(a)), indicating that XY03-EA reduced the autophagy flux in the PC-12 cells induced by OGD/R. XY03-EA enhanced the cell viability of I/R injury, scavenged ROS, and inhibited autophagy flow, suggesting that XY03-EA inhibited autophagy-induced cell death in I/R injury. These findings are consistent with studies by Wang and Xu [38], Liu et al. [39], and Sun and Yue [40] in reducing autophagic cell death from I/R injury through inhibiting oxidative stress.

As mentioned above, XY03-EA reduced the autophagy flux by blocking the autophagosome and lysosome fusion (Figure 9(a)). It is reported that the fusion process is carefully regulated by the Atg4 redox state. Atg4 is a cysteine protease, as a conjugating and deconjugating enzyme, regulating the essential esterification process of LC3 during autophagy [41]. Under ischemic situations, the increased mitochondrial oxidative stress leads to the inactivation of Atg4 at the site of autophagosome formation. As the autophagosome fuses with the lysosome, Atg4 is locally reactivated to delipidate and recycle LC3 [42]. When I/R injury occurs, Atg4 levels are upregulated and autophagy is activated [43]. A recent study shows that resveratrol prevents the generation of ROS induced by dopamine and protects neurocytes by restoring the level of Atg4 [44]. We supposed that XY03-EA regulated the expression of Atg4 and impacted autophagosome maturation and the subsequent autophagy resolution. Compared with the expression of Atg4 in the PC-12 cells induced by OGD/R, XY03-EA inhibited the Atg4-mediated autophagosome formation and protected the neurocytes from ROS injury.

Although the mechanisms underlying the balance between ROS-induced autophagic protection and autophagic cell death remain unclear, studies have found that various signaling pathways have been reported to be involved in the interaction between ROS and autophagy, including mTORC1-ULK1 [45], AMPK-mTORC1-ULK1 [46], and mitophagy. In this study, we found that when OGD/R injury occurred, the AMPK pathway was activated, and the AMPK pathway negatively regulated the mTOR pathway and activated the downstream ULK1 and Beclin 1. Notably, after XY03-EA intervention, AMPK signaling pathway activation was inhibited, mTOR was activated, the expression of ULK1 protein and Beclin 1 subsequently was decreased, and Bcl-2 expression was increased (Figures 9(b)–9(f)). These results suggested that XY03-EA inhibited the initial stage of autophagy through AMPK-mTORC1-ULK1 and Bcl-2-Beclin 1 signaling pathways. In combination, these findings suggested that XY03-EA play dual roles in the regulation of autophagy by impeding the initiation of autophagy and the completion of autophagy flux.

**Figure 10:**
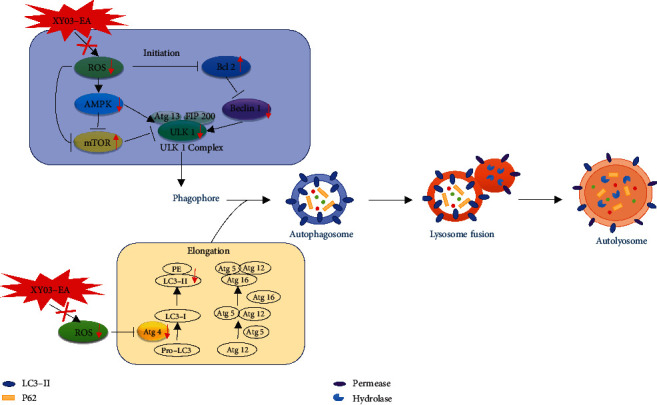
Mechanism diagram of XY03-EA protecting I/R injury

In this study, we provided solid evidence that XY03-EA could protect the brain tissue from cerebral I/R injury in rodents and nonhuman primates. We found that ROS-mediated oxidative stress injury indeed caused excessive activation of autophagy, which if unrepaired caused autophagic cell death. The autophagic cell death caused by excessive ROS was inhibited by XY03-EA by scavenging ROS. Mechanistically, XY03-EA appeared to inhibit the initiation and elongation of autophagy through AMPK-mTORC1-ULK1 and Bcl-2-Beclin 1 signaling pathways and the formation of autophagosome and autolysosome (Figure 10). Notably, in addition to ROS-mediated autophagy neurocyte death, several pathways of cell death are involved including apoptosis and pyroptosis. XY03-EA, as a potent antioxidant, needs further researches to explore the underlying mechanism in detail.

## Figures and Tables

**Table 1:** The criteria of neurological deficit score in monkeys.

Category	Score
(1) State of consciousness	
Normal, always alert	0
Conscious and aggressive	4
Conscious, can avoid stimulus	6
Conscious, sometimes unresponsive to stimulus	8
Sleepy, stimulation can wake	10
Comatose, can open eyes to short and strong stimulus	16
Comatose, need to continue strong stimulation to be awakened	20
Shallow coma, pupil to light reflection exists	24
Deep coma, the pupil to light reflection disappeared	28
(2) Motor system	Healthy/affected
Movement intensity of upper limb	
Normal	0/0
Less	2/2
Paralysis	4/4
Movement intensity of lower limbs	
Normal, moving freely	0/0
Lift legs and bend knees	2/2
Need help to move	4/4
Paralysis	6/6
Upper limb tension	
Normal	0/0
Complete spasm or relaxation	3/3
Lower limb tension	
Normal	0/0
Complete spasm or relaxation	3/3
(3) Skeletal muscle coordination	
Walk normally	0
Slightly uncoordinated, with gait distortion when walking	4
Incongruous, but climbable	6
Can stand but fall down after a few steps	10
Can sit, but cannot walk	12
Lie on your side or back only	16
No movement	18
(4) Sensory system	Healthy/affected
Face feeling	
All regions responded to contact stimulus	0/0
Some areas do not respond	3/3
Ear reaction	
An incitement response to a stimulus	0/0
Lack of response to stimulus	3/3
Pain response	
Styling the toes can produce a strong, rapid, and complete foot shrinkage response	0/0
Response to diminished	3/3
Response to disappear	5/5

**Table 2:** Pharmacokinetic parameters of XY03-EA on MCAO/R rat whole blood.

PK parameters^∗^	25 mg/kg (p.o.)	50 mg/kg (p.o.)	100 mg/kg (p.o.)
	XY03-EA	XY03-EA	XY03-EA
*C*_max_ (nM)	2710 ± 4569	2647 ± 2924	14174 ± 16787
*T*_max_ (h)	0.750 ± 1.35	0.646 ± 1.355	0.396 ± 0.648
AUC_0-4h_ (nM∙h)	1672 ± 1633	2022 ± 1467	6960 ± 7170
*t*_1/2_ (h)	5.01 ± 7.38	3.91 ± 2.84	1.82 ± 1.13
MRT (h)	6.44 ± 10.2	4.59 ± 3.60	1.65 ± 0.854

^∗^ *C*_max_: maximum plasma concentration; *T*_max_: time to maximum plasma concentration; AUC_(0-4h)_: area under the curve (from 0 h to 4 h); *t*_1/2_: terminal half-life; MRT: mean residence time.

**Table 3:** The NDS progression rate of monkeys after the MCAO/R.

Group	MCAO/R	XY03-EA 25 mg/kg	XY03-EA 50 mg/kg
Day	Mean ± SD	Mean ± SD	Mean ± SD
D21:D1	6 ± 26	−89 ± 23^#^	−68 ± 46^#^
D14:D1	28 ± 36	−81 ± 26^#^	−62 ± 40^#^
D7:D1	13 ± 34	−62 ± 25^#^	−37 ± 33
D3d:D	41 ± 33	−51 ± 22^#&^	−14 ± 17^#^

The rate of NDS progression (%) = (score − score_D1_)/score_D1_∗100%.^#^*p* < 0.05 vs. the MCAO/R group; ^&^*p* < 0.05 vs. the NBP group.

## Data Availability

All datasets generated for this study are included in the manuscript file.

## Abbreviations

AMPK: Adenosine 5′-monophosphate-activated protein kinase
ANOVA: One-way analysis of variance
Atg: Autophagy-related proteins
CBF: Cerebral blood flow
CCA: Common carotid artery
DMEM: Dulbecco's Modified Eagle's Medium
DSA: Digital subtraction angiography
DTI: Diffusion tensor imaging
ECA: External carotid artery
FBS: Fetal bovine serum
FOV: Field of vision
GSH: Glutathione
ICA: Internal carotid artery
IL: Interleukin
LC3: Microtubule-associated protein-1 light chain 3
LD: Lactic acid
MOA: Mechanism of action
MCAO/R: Middle cerebral artery occlusion and reperfusion
MDA: Malondialdehyde
MRI: Magnetic Resonance Imaging
mTOR: Mammalian target of rapamycin
NBP: L-3-n-Butylphthalide
NC: Nitrocellulose filter
NDS: Neurological deficit score
OA: Occipital artery
OGD/R: Oxygen-glucose deprivation and reoxygenation
PBS: Phosphate buffer
PMSF: Phenylmethylsulfonyl fluoride
PE: Phosphatidylethanolamine
RMCA: Right middle cerebral artery
ROS: Reactive oxygen species
SOD: Superoxide dismutase
ST: Superior thyroid artery
T1WI: T1-weighted imaging
T2-FLAIR: Liquid attenuation inversion recovery
T2WI: T2-weighted imaging
TE: Echo time
TNF: Tumor necrosis factor
TR: Repeat time
TTC: 2, 3, 5-Triphenyltetrazolium chloride
ULK1: UNC-51-like kinase 1
ZO-1: Zonula occludens-1

## Supplementary Materials

Supplementary Materials Supplementary data 1: XY03-EA had certain scavenging effect on oxygen free radicals.
