# Supplementary material for: Inhibition of Autophagy Facilitates XY03-EA-Mediated Neuroprotection against the Cerebral Ischemia/Reperfusion Injury in Rats
Source: Oxid Med Cell Longev. 2022 Mar 30;2022:7013299. doi: 10.1155/2022/7013299 (PMC8986424; doi:10.1155/2022/7013299)
Supplement: Supplementary Materials — Supplementary data 1: XY03-EA had certain scavenging effect on oxygen free radicals. [file 7013299.f1.doc]

Supplementary data 1：XY03-EA had certain scavenging effect on oxygen free radicals

Methods: The free radical scavenging effect of XY03-EA was detected by electron spin resonance technique (ESR). The specific method is briefly described as follows: The signal intensity of OH· adduction produced by spin capture of Fenton reaction by 5, 5-Dimethyl-1-Pyrrolidine-n-oxide (DMPO, a free radical trapping agent) was detected by electron para-magnetic resonance (EPR) spectrometer (Bruker EPS300, German). The free radical scavenging efficiency of the tested compound was calculated according to the change of EPR spectrum intensity before and after the addition of the tested compound. The final concentration of all samples was about 100μg/ mL.

Scavenging Efficiency (%) = (h_0_-hx)/h_0_*100%

Results: The scavenging efficiency of Edaravone and XY03-EA were 52.742% and 13.653% respectively. XY03-EA had certain scavenging effect on oxygen free radicals.

| Compound | h_0_ | h_0_-h_x_ | Scavenging Efficiency (%) |
| --- | --- | --- | --- |
| Blank | 72221 | 0 | -- |
| Edaravone | 34130 | 38091 | 52.742 |
| XY03-EA | 62361 | 9860 | 13.653 |
